# Supplementary material for: Are carbon emissions trading and green financial instruments synergistic? -Comprehensive quantitative research based on content analysis
Source: PLoS One. 2024 Mar 7;19(3):e0298601. doi: 10.1371/journal.pone.0298601 (PMC10919601; doi:10.1371/journal.pone.0298601)
Supplement: S1 Data — (ZIP) [file pone.0298601.s001.zip › Data/地级市Z_F工作报告.zip!/╜¡╦╒/╒≥╜¡╩╨/└┤╫╘╣1⁄2╓┌║┼í╛┬φ┐╦╩2╛▌═°í┐_2009.docx]

2008年工作回顾

　　过去的一年，是我市发展进程中很不平凡的一年，国际国内经济环境复杂多变，面临的挑战极其严峻，经受的考验非同寻常。我们在市委的坚强领导下，坚持以科学发展观为指导，以“推进思想解放、推动科学发展”为主线，以“做人民群众喝彩的事”为目标，与全市人民一道，攻坚克难、开拓进取，基本完成市六届人大一次会议确定的各项目标任务。

　　（一）国民经济平稳增长，运行质量有所提升

　　预计完成地区生产总值1421亿元，同比增长13.6%，人均超过6700美元。财政总收入233.2亿元，增长14.7%，其中地方财政一般预算收入85.66亿元，增长6.7%，完成市人大常委会调整指标。全社会固定资产投资718.5亿元，增长22.2%。

　　以高效农业规模化为重点，推动现代农业优化布局、调整结构。优化“三大板块”、“七条走廊”的区域布局，新增高效农业面积20.88万亩，其中设施农业5.44万亩，新增农村“三大合作”组织240家、农业“三品”认证52个，有力促进了农业增效、农民增收。

　　以打造千亿产业、百亿企业为重点，推动工业集群发展、高端延伸。装备制造、新材料、绿色化工、特种金属和造纸五大产业实现销售超2000亿元，增长32%，占全市工业总量的80%。其中，装备制造业实现销售1050亿元，率先突破千亿目标。新增两家上市公司，两个投资超10亿元的重大项目成功落户。

　　以实施现代服务业三年翻番行动计划为重点，推动服务业规模扩张、层次提升。现代物流业等九大服务行业实现营业收入1137亿元，增长24%。社会消费品零售总额411亿元，增长24%，创近年来新高。

　　（二）城乡建设奋力推进，基础设施日益完善

　　突出规划龙头地位，促进城乡资源要素集约利用。编制完成全市电力、通讯、绿地、污水处理系统等一批重大基础设施规划和丁卯-三山地区战略发展规划，区域发展空间布局进一步优化。

　　破解城乡拆迁难题，城建重点工程有序推进。坚持依法、阳光、惠民、和谐拆迁，完成拆迁面积310万平方米，其中市区175万平方米，创历史新高。南徐新城道路骨架初现雏形，规划展示馆顺利封顶。金山广场、西津渡历史文化街区保护更新二期工程建成并对公众开放，金山湖整治、引航道水利枢纽和焦南闸扩建工程进展顺利。

　　加大建设力度，综合交通能力不断增强。交通基础设施投入超过60亿元，投资总额为历年最高。“两铁”拆迁进度全线领先，泰州大桥南接线、镇大公路开工建设。沿江高等级公路完成80%的工程量，243省道、宁杭高速公路镇江段等重点工程相继建成。全社会港口吞吐量突破亿吨，其中沿江港口达8700万吨。

　　（三）改革开放继续深入，发展活力明显增强

　　对外开放水平进一步提升。实际利用外资12.1亿美元，增长13.7%。太阳能光伏、船用装备、新材料等产业招商取得重大突破。开发园区实际利用外资占全市比重达75%，同比提高15个百分点。完成进出口总额73.6亿美元，增长16.6%，其中出口42亿美元，增长13.9%；完成外经营业额2.56亿美元，增长24.4%。

　　体制机制创新成效明显。国企改革进一步推进。市属事业单位分类改革有序推开。制订新一轮财政体制调整方案。在全国率先试点“淡马锡”中小企业融资模式，全省首家农村小额贷款公司挂牌营业，另有6家正在筹建。新增民营企业3700家，个体工商户13800户，到位民资175亿元。

　　（四）生态建设力度加大，环境质量不断改善

　　确立2010年创建国家生态市的目标定位，节能减排成效明显。完成国家生态市建设规划修编，扎实推进生态县（市）和环境优美乡镇创建工作。化工集中区压减为3个，扬中、句容和京口、润州不再设立化工集中区；关闭小化工企业25家、电镀企业37家。黄岗取水口延伸工程顺利推进。化学需氧量、二氧化硫排放量分别削减2.2%和4%，单位GDP能耗下降4.82%。市区空气质量良好以上天数达332天。

　　强力推进太湖流域治理，水环境整治初见成效。太湖流域内19个乡镇的污水处理厂开工建设，199家工业企业提标改造，对入湖河道实施综合治理、重点污染源实行在线监测，水环境整治工作取得初步进展。

　　（五）加强统筹协调，推动经济社会和谐发展

　　全力改善民生。认真落实市委2008年1号文件精神，以辖市为单位达到全面小康省定目标值。城镇居民人均可支配收入19040元，增长13.5%；农民人均纯收入8742元，增长14%。城镇登记失业率为2.54%，各类社会保险参保覆盖率进一步提高。15件为民办实事顺利完成，农村新五件实事全部办结。

　　推动各类社会事业较快发展。县级区域教育现代化创建基本达到省定标准。全面启动《引进培育创新创业领军人才三年行动计划》。成功举办市第十三届运动会、金山旅游文化节、首届“文化嘉年华”等群众性文体活动，“文心公益行动”深受市民欢迎，新广电中心顺利封顶。市第一人民医院内科医技大楼开工建设。成为全国城乡居民医疗保险相衔接试点地区。食品药品安全和安全生产工作进一步加强。

　　切实加大经济工作组织力度。面对金融风暴的巨大冲击，各级政府迅速反应，与企业共同应对各种困难。兑现各项税费优惠政策，共取消、减免行政事业性收费和基金项目87项，综合费率由4％。下降至1％。

　　2009年主要工作

　　2009年是建国60周年，也是实施“十一五”规划的关键一年。当前，全球金融危机还在蔓延，宏观经济环境短期内难以根本好转，企业运行困难的局面仍将持续，政府组织经济工作的压力依然很大。但挑战是相同的，困难是共性的，只要我们坚定信心、积极应对，迎难而上、奋力作为，就一定能实现镇江发展的新跨越！　 2009年全市经济社会发展的主要预期目标是：地区生产总值增长11%；地方财政一般预算收入增长13%；全社会固定资产投资增长20%；社会消费品零售总额增长18%；实际利用外资增长5%，进出口总额增长5%；城镇居民人均可支配收入增长12%，农民人均纯收入增长12%；城镇登记失业率控制在4%以内；单位GDP能耗下降4.4%，化学需氧量、二氧化硫排放量分别削减2.2%和4%。工作中，我们将力争做到主要经济指标增幅高于全省平均水平、快于全省沿江八市。

　　围绕上述目标，我们将全力抓好以下六个方面：

　　（一）努力保持经济平稳较快发展

　　保持经济平稳较快发展是政府工作的首要任务，我们将以项目建设为主要抓手，以大项目带动大投入、大调整，全面增强投资、消费和出口的拉动力，确保实现“保增长、扩内需、调结构”的目标。

　　扩大有效投入。力争完成全社会固定资产投资1000亿元，其中产业类投资600亿元以上。坚持扩大投资规模和优化投资结构并举，重点投向“千百亿工程”，积极推进一批先进制造业、新能源、新技术重点项目，五大产业力争实现销售收入2600亿元，占工业总量的85%。加快高新技术产业化和传统产业高新化步伐，确保高新技术产业产值突破1000亿元。继续实施现代服务业三年翻番行?项目。以大港港口、惠龙钢铁和中储粮等为重点，做大现代物流产业；以省级大学科技园、软件园、新区国际服务外包示范区等为重点，做强科技信息产业。力争服务业增加值占GDP的比重达39%以上。吸引“三资”投入农业34.5亿元，完成土地流转10万亩，发展“三大合作”组织300家，新增高效农业20万亩。

　　增加即期消费。城区内建设一批高档商场、宾馆、品牌店和休闲娱乐设施，促进本地消费。积极拓展农村消费市场，实现现代流通网络镇村两级全覆盖。大力实施24个总投资1000万元以上的旅游重点项目，提档升级“三山”风景区，力争实现旅游总收入270亿元。促进房地产市场健康发展，开工建设经济适用住房10万平方米、廉租房1.1万平方米、安居房90万平方米、商品房200万平方米，改善2万户以上居民居住条件。

　　稳定外贸出口。抓住国家大幅上调出口退税率的机遇，鼓励外贸企业加快发展，推动外贸产品转型升级。

　　（二）奋力突破体制机制瓶颈

　　加大重点领域和关键环节的改革力度，破解发展难题，增强发展活力。

　　深化投融资改革。坚持先规划后建设、先征地后配套、先储备后开发、先做环境后出让，实现城市资产效益最大化。进一步加大市城投公司等六大投融资平台建设力度，增强政府投融资能力，力争总资产达到400亿元。改善金融服务，积极引进外资和股份制银行，新增农村小额贷款组织7家以上，力争全市贷款余额突破1200亿元，着力破解中小企业融资难题。

　　推进其他领域改革。积极推动农村改革和制度创新，培育农民新型合作组织，加快农村土地流转。全面完成国有企业改革扫尾工作。开展新一轮机构改革，严控机构编制增长。全面推进事业单位分类改革。落实财政管理体制改革方案。

　　（三）加快推进城乡一体化建设

　　深入贯彻落实党的十七届三中全会精神，发挥好中心城市对农村的辐射带动作用，着力构建城乡经济社会一体化发展新格局。

　　建设花园城。南徐新城区力争规划展示馆竣工对外开放，新行政中心办公楼主体工程封顶，总投资14.74亿元的体育会展中心开工建设，加快推进中心商务区建设，基本形成新城区道路框架，基本完成安居房建设，南山景区西大门及广场建成并投入使用，初显花园新城雏形。

　　打造第一景。加快北部滨水区建设，全景开放金山湖景区、全线贯通滨江旅游专线、全面完成内江控水主体工程、全面启动内江清淤工程、全部封顶金泉花园安置房。坚持高标准、大力度出新长江路沿线，打造西津渡品牌。

　　完善大交通。加快京沪高铁、沪宁城际铁路镇江段及场站等配套工程建设，规划实施以“两铁”站为节点的综合交通枢纽，推进宁杭城际铁路镇江段以及泰州大桥南接线建设，启动大港四期工程，建成兴隆港一期工程；完成镇大公路拓宽改造工程，做好五凤口高架前期准备，建成禹山北路东段、左湖路，形成连接主城与镇江新区的两条主干道。

　　改造老城区。继续加大拆迁改造力度，全市完成拆迁260万平方米，形成若干新的城市商务区。实施中华路、双井路、迎江路、新河街片区等50万平方米危旧房片区改造，推进40万平方米老小区整治，投入8亿元改造庄泉村、戴家湾、新华村等城中村。投入3亿元实施高速公路镇江西、镇江南和镇江出入口环境综合整治。

　　统筹城乡建设。

　　（四）切实加大生态建设力度

　　紧扣国家生态市建设指标体系，实行最严格的环境保护制度，以刚性约束保护生态环境。

　　加快生态市创建步伐。全面实施《镇江生态市建设规划纲要》，确保实现国家生态市建设的阶段性目标。

　　加强太湖流域水污染治理。建成投运太湖流域19个乡镇污水处理设施。

　　加大绿化造林力度。积极创建国家生态园林城市。新增绿化造林15.6万亩，全市森林覆盖率达到23.9%。

　　（五）着力改善城乡居民生活

　　大力扶持创业。把全民创业作为城乡居民增收的根本途径，落实激励政策，优化创业环境。

　　积极促进就业。实行更加积极的就业政策，一手抓扩大就业，一手抓控制失业。深入实施“充分就业市”创建三年行动计划。完善就业服务体系，开展职业技能培训2.5万人次。新增城镇就业3万人，下岗失业人员再就业1.5万人，农村劳动力转移3万人。

　　切实完善保障。加快推进城乡社会保障制度全覆盖，企业职工基本养老保险新增扩面3万人，新型农村养老保险和城乡居民养老保险参保覆盖率超过50%。推进市与辖市医保政策接轨，城乡居民医保人口覆盖率稳定在90%以上。高度重视慈善事业，完善城乡救助体系。消除年人均收入2500元以下的贫困人群，消除年集体收入10万元以下的行政村。

　　（六）协调推进各项社会建设

　　坚持以人为本的发展理念，进一步加大社会事业领域的改革和投入力度，促进社会繁荣和谐。

　　提高全民素质。加大争创全国文明城市工作力度，大力弘扬新时期镇江精神，以“大爱镇江”特色活动为主线，深化“百万市民学文明礼仪”系列活动，广泛开展“我与文明同行”等群众性道德实践活动，深入开展“和谐社区”、“和谐家庭”等创建活动，切实提升城市软实力，进一步形成良好社会风尚。

　　加强文化建设。加大项目建设力度，形成一批具有带动产业链发展的龙头企业和具备承载产业发展功能的文化产业园区。建设西津渡民俗文化街区和特色博物馆区，打造健康路健身文化休闲区，建成金山宝地大型演艺广场，提升大市口商业文化娱乐圈，兴建新广电中心影视文化城，创作有市场、有前景、有重大影响的文化艺术精品。继续开展“文化嘉年华”系列活动，免费开放市图书馆。

　　繁荣社会事业。加快科教兴市进程，力争第七次创成全国科技进步先进市。坚持公共医疗卫生的公益性质，强化政府责任和投入，加快推进市第一人民医院内科医技楼、中医院门诊外科综合大楼建设，迁建市第二人民医院和中心血站。新建全民健身点50个。六创全国双拥模范城。争创节水型城市。

　　维护社会稳定。制订应急预案，着力防范企业生产经营风险，妥善处置企业裁员、拖欠工资等问题，避免引发群体性事件。强化食品、药品监管，创建“质量兴市先进市”。加强社会治安综合治理，增强人民群众的安全感。切实加强信访工作，发挥“大调解”机制作用，确保社会和谐稳定。

　　努力建设人民满意的现代服务型政府

　　面对严峻形势，承载人民重托，市政府全体组成人员和公务员，将以时不我待的紧迫感，树立现代服务理念，按照现代政务方式建设服务型政府，使政府工作充满生机和活力。

　　强化责任意识。对人民负责是政府工作的基本准则。在困难的形势下，全市各级政府、全体公务人员将强化为基层服务的责任、抓工作落实的责任，与企业、与群众共渡难关，多做人民群众喝彩的事。全面实施行政问责制，强化督查、行政监察、审计监督和绩效评估，做到有责必问、有错必究。

　　坚持依法行政。遵守法律是政府工作的根本原则。深入推进法治政府建设，全面提高依法行政能力。自觉接受市人大及其常委会的依法监督，主动接受市政协和各民主党派、工商联、无党派人士的民主监督。加强政务公开，重大决策实行社会参与，保障人民群众的知情权、参与权、表达权和监督权。

　　提高行政效能。效能是政府管理的核心要素。以新一轮政府机构改革为契机，以提高行政效能为突破口，整合行政机构，简化审批事项，改进工作方式，使机关运转更加快捷高效。加强电子政务建设，努力运用现代方式优化工作流程，提高行政效率。

　　保持清正廉洁。清廉是政府机关的第一形象。深入落实廉政建设责任制，建立健全惩防体系，全面推进行政权力网上公开透明运行。坚决反对铺张浪费，带头过紧日子，严格控制出国出境、公务接待和公车使用等支出。全面实施市直部门收支脱钩、零基预算，切实降低行政成本。
